# A Feedback Loop Driven by H3K18la and ASF1B via the LINC02732-miR-1291 Axis Promotes Hepatocellular Carcinoma Proliferation

**DOI:** 10.3390/cells15100952

**Published:** 2026-05-21

**Authors:** Jingya Yu, Lulu Xin, Ying Cui, Chunxin Fan, Yongheng Yang, Xiaolu Zhang

**Affiliations:** 1 Department of Genetics, School of Basic Medical Sciences, Cheeloo College of Medicine, Shandong University, Jinan 250012, China; 2 Department of Physiology and Pathophysiology, School of Basic Medical Sciences, Cheeloo College of Medicine, Shandong University, Jinan 250012, China

**Keywords:** HCC, H3K18la, ASF1B, ncRNA

## Abstract

**Highlights:**

**What are the main findings?**

- H3K18la enrichment on LINC02732 promoter influences LINC02732-miR-1291-ASF1B in hepatocellular carcinoma.
- ASF1B promotes H3K18la enrichment via p300 recruitment, constituting a mutual positive regulatory feedback between H3K18la and ASF1B.

**What are the implications of the main findings?**

- Histone lactylation modulates the ncRNA regulatory network, and they cooperatively facilitate the progression of hepatocellular carcinoma.

**Abstract:**

Histone lactylation acts as a master regulator in tumor development, but its role in a noncoding RNA (ncRNA) network remains unclear. This study aims to reveal the interaction between H3K18la and the lncRNA-miRNA-mRNA regulatory network in hepatocellular carcinoma (HCC). Transcriptome sequencing and ChIP sequencing were performed in HCC and adjacent normal tissues. Cut&Run and qPCR were used to validate the H3K18la enrichment on LINC02732 and CD44 promoter. Dual luciferase reporter assay, qPCR and Western blotting were used to verify the LINC02732-miR-1291-ASF1B axis. Co-Immunoprecipitation was performed to validate ASF1B recruiting p300. CCK8 and mouse subcutaneous tumor formation were performed to demonstrate this axis promoting HCC. H3K18la enrichment on LINC02732 promoter elevates its expression in both HCC samples and cell lines, therefore enhancing ASF1B expression via sponging miR-1291. Moreover, ASF1B, a histone chaperone, promotes H3K18la by recruiting lactyltransferase p300, forming an ASF1B-H3K18la positive feedback loop. The axis upregulates CD44 expression and promotes HCC in vitro and in vivo. These findings demonstrated the influence of H3K18la on the LINC02732-miR-1291-ASF1B axis and the novel role of ASF1B in histone lactylation by recruiting p300, which together promoted HCC proliferation.

## 1. Introduction

In 2022, HCC was the sixth most common cancer and the third leading cause of cancer-related deaths worldwide. Moreover, the incidence was estimated to increase in the next 30 years [1,2]. Since the liver is an important metabolic organ, its malignant transformation can cause the abnormal accumulation of various substances, including lactate. A lactate-related post-translational modification, called Lysine lactylation (Kla), has been recently discovered, in which a lactyl moiety is transferred onto the ε-amino group of lysine residues [3]. Kla was identified in both histone and non-histone proteins, with p300 working as a Kla “writer” (lactyltransferase), especially for histones [4,5]. Aberrant Kla patterns are linked to various diseases, including metabolic disorders, inflammation, and cancer [3,4,6]. Histone lactylation, as an important epigenetic modification, was observed in H2A, H2B, H3, and H4 [3]. Among them, H3K18la is one of the most studied sites. In septic shock patients, H3K18la level is positively correlated with serum IL-6 and IL-10 levels [7]. In colorectal cancer, H3K18la promotes bevacizumab resistance by facilitating the expression of the autophagy enhancer protein RUBCNL [8]. The O-GlcNAcylation of YBX1, a protein associated with glycolysis, promotes HCC development by upregulating H3K18la [9]. Single-cell sequencing revealed that hepatic stellate cells promoted HCC development by regulating histone lactylation, including H3K18la [10]. SRSF10/glycolysis/H3K18la formed a positive feedback loop that promotes M2 macrophage polarization, thereby inhibiting CD8^+^ T cell activity and decreasing the efficacy of anti-PD-1 therapy in HCC [11].

Histone chaperones play an important role in various chromatin epigenetic modifications, including acetylation, methylation, and phosphorylation. Anti-silencing Function 1 (ASF1), which works as a histone chaperone for H3-H4 histones, involves two paralogs: ASF1A and ASF1B. ASF1 plays critical roles in chromatin assembly, DNA replication and repair, and cell cycle regulation [12]. ASF1 can partially dissociate nucleosomes and expose the N-terminal tails of histones H3 and H4 that were previously masked, thereby facilitating histone acetyltransferases (HATs), such as p300/CREB-binding protein (CBP). Additionally, ASF1 can recruit HATs to specific histones [13]. Although the two paralogs ASF1A and ASF1B share a highly similar amino acid sequence, their expression pattern and function are not exactly the same. ASF1A is widely expressed in various cells, mainly playing a role in DNA repair; therefore, it serves as an antitumor agent in certain cancers [14]. ASF1B has a specific expression in proliferative cells and promotes the progression of multiple cancers including HCC [15,16,17]. ASF1A can increase H3K18la level by recruiting p300, thereby promoting atherosclerosis via Endothelial-to-Mesenchymal Transition [18]. However, whether ASF1B plays a role in H3K18la regulation remains unclear.

The regulatory role of H3K18la in the expression of various mRNAs was previously revealed. Besides mRNAs, the significance of non-coding RNAs (ncRNAs) has also been recently recognized. microRNAs (miRNAs), with transcripts of approximately 22 nucleotides, can target complementary mRNA, resulting in degradation or translation repression. Long noncoding RNAs (lncRNAs), with a length greater than 200 nucleotides, can competitively bind to miRNA response elements, therefore decreasing miRNA-induced gene silence. The cross-regulation between lncRNAs, miRNAs, and mRNAs forms a complicated regulatory network, which plays a comprehensive role in gene expression and cancer progression [19,20]. Nevertheless, how H3K18la influences lncRNA expression and the ncRNA regulatory network is still unclear. Accordingly, this study explored how H3K18la influences the ncRNA network via the regulation of lncRNA expression in HCC using the combination of ChIP-Seq and lncRNA/miRNA/mRNA sequencing and demonstrated the function of the H3K18la-LINC02732-miR1291-ASF1B axis. This study further revealed that ASF1B can facilitate H3K18la by recruiting p300, thereby forming a positive feedback loop and promoting HCC proliferation by stimulating CD44 expression.

## 2. Materials and Methods

### 2.1. Patients with HCC

Twenty-two patients with newly diagnosed histologically confirmed HCC were recruited from Shandong University Qilu Hospital, China. Surgically resected tumor and paired adjacent normal (AN) tissues were obtained from the patients. Histopathological assessment was used to validate tumor components in collected samples, and all qualified specimens were frozen at −80 °C prior to use. Ethical approval was granted by the Ethics Committee of Shandong University, China, with an ethics code SDULCLL2021-1-27 approved on 19 November 2021. Each participant provided signed written informed consent, and the present study fully adhered to the Declaration of Helsinki. Among all patient samples, the Sequencing cohort samples were used for high-throughput whole genome expression study while the Validation cohort samples were used for sequencing data validation.

### 2.2. RNA Sequencing

Total tissue RNA extraction was performed by using the TRIzol reagent kit (Life Technologies, Carlsbad, CA, USA). RNA integrity and purity were assessed on 1% agarose gels under the NanoPhotometer^®^ spectrophotometer (IMPLEN, Westlake Village, CA, USA). First-strand cDNA was synthesized from 3 μg of total RNA with M-MuLV reverse transcriptase (RNase H) and random hexamer primer. NEB 3′ SR adaptor was ligated to the 3′ end of miRNA, and the SR RT primer converted the single-stranded DNA adaptor into a double-stranded DNA fragment. Subsequent PCR amplification was performed by universal PCR primers, index (X) primer and Phusion high-fidelity DNA polymerase. PCR products were purified with AMPure XP system, and the sequencing library was constructed by using the NEBNext^®^ UltraTM RNA library prep kit, normalized to a concentration of 10 pM, and sequenced on HiSeq X Ten (Illumina, San Diego, CA, USA) at Novogene Science and Technology Co., Ltd. (Beijing, China). Raw sequencing data were filtered to remove adapter-contaminated reads, poly-N-containing reads and low-quality sequences. FPKM of each gene was calculated according to the length of the gene and read count mapped to this gene. Differential expression analysis was conducted using the DESeq2 R package (1.10.1). Functional enrichment analyses of differentially expressed (DE) genes, including Gene Ontology (GO) and Kyoto Encyclopedia of Genes and Genomes (KEGG) pathway analysis, were performed by using the clusterProfiler R package 4.14.3.

### 2.3. Chromatin Immunoprecipitation Followed by High-Throughput Sequencing (ChIP-Seq)

ChIP-Sequencing was performed by Novogene Science and Technology Co., Ltd. (Beijing, China). Cells were cross-linked with 1% formaldehyde for 10 min at room temperature, and the reaction was quenched with glycine (final concentration 0.125 M). Cells were lysed, and chromatin was sheared into 200–500 bp fragments using a sonicator. The sheared chromatin was immunoprecipitated overnight at 4 °C with specific antibody against the H3K18la or IgG (PTM BioLab, Hangzhou, China). Immunocomplexes were captured by protein A/G magnetic beads, washed sequentially with low-salt, high-salt, LiCl-containing, and TE buffers, then eluted with elution buffer. Cross-links were reversed at 65 °C overnight, and DNA was purified using phenol-chloroform extraction and ethanol precipitation. The purified DNA libraries were constructed using a DNA library prep kit, quantified by agarose gel electrophoresis, and sequenced on an Illumina NovaSeq platform.

### 2.4. HCC Cell Lines and Cell Culture

The HCC cell lines PLC/PRF/5 and Huh-7 were purchased from the Stem Cell Bank, Chinese Academy of Sciences (Shanghai, China). These cells were cultured in MEM/DMEM medium (Thermo Fisher Scientific, Waltham, MA, USA) containing 10% fetal bovine serum, 2 mM L-glutamine and 100 units/mL penicillin. SiRNAs targeting ASF1B and LINC02732 were purchased from (GENCEFE Biotech, Wuxi, China). The sequences were Si-ASF1B-1: GGUUCGAGAUCAGCUUCGATT, Si-ASF1B-2: GCUACUACGUCAACAACGATT, Si-LINC02732-1:GCUACAGAUGAUCUUACAATT, Si-LINC02732-2:AGUGCUAUGUGUCUAGCUATT. Cells were transfected with siRNAs using Lipofactamine 3000 (Thermo Fisher Scientific) according to the protocol provided. Cells were harvested for analyses 72 h post-transfection. ASF1B over-expression lentivirus was purchased from Genechem (Shanghai, China).

### 2.5. Quantitative PCR (qPCR)

Total RNA in cells and tissues was extracted using Trizol (Life Technologies, Carlsbad, USA). cDNA was synthesized using SuperRT One Step RT-PCR Kit (Jiangsu CoWin Biotech, Taizhou, China). qPCR was carried out in a Roche LightCycler 96 sequence detector (Roche, Basel, Switzerland) using SYBR Green and specific primers. GAPDH expression was used as a control for lncRNA, circRNA and mRNA. U6 expression was used as a control for miRNA. The relative expression levels of target RNAs were quantified using cycle threshold (Ct) values and normalized to the endogenous reference genes GAPDH or U6. All qPCR primer sequences are summarized in Table S1.

### 2.6. Western Blot Analysis

Total protein was extracted with RIPA Buffer (Thermo Scientific, Waltham, MA, USA) supplemented with 1% Phenylmethanesulfonyl fluoride (Millipore Sigma, Burlington, MA, USA). Protein concentration was determined using the DC Protein Assay (Bio-Rad, Hercules, CA, USA). Equal amounts of protein (30 μg) were separated via electrophoresis on Mini-PROTEAN TGX Gels (Bio-Rad) and electrotransferred onto PVDF membranes using the Trans-Blot Turbo Transfer System (Bio-Rad). Membranes were blocked with 5% non-fat milk and then incubated with corresponding primary antibodies and secondary antibodies. Protein bands were visualized using Clarity Max Western ECL Substrate (Bio-Rad, 1705062) and captured with the ChemiDoc MP Imaging System (Bio-Rad). The primary antibodies utilized in this study were as follows: ASF1B, β-actin and CD44 (ABclonal Technology, Wuhan, China); p300 (Proteintech, Rosemont, IL, USA); and H3 and H3K18la (PTM BioLab).

### 2.7. The Dual Luciferase Reporter Assay

The LINC02732 and ASF1B reporters containing wild-type/mutant miR-1291 pairing sequence were constructed from Boshang Biotech (Jinan, China). The promoter plasmids were transfected into cells together with the control or miR-1291 mimic. Cells were harvested 48 h after transfection, and luciferase activity was measured using the Dual-Luciferase Reporter Assay System (Promega, Madison, WI, USA). Firefly luciferase signals were normalized to Renilla luciferase activity.

### 2.8. Co-Immunoprecipitation

Cells were harvested and lysed in ice-cold RIPA buffer supplemented with protease and phosphatase inhibitors. The cell lysate was centrifuged at 12,000× *g* for 15 min at 4 °C to remove insoluble debris. The supernatant was incubated with a specific antibody against the target protein or IgG (as negative control) overnight at 4 °C with gentle rotation. Protein A/G magnetic beads were added to the mixture and incubated for 2 h at 4 °C. The beads were washed 4 times with cold RIPA buffer, and the immunocomplexes were eluted by boiling in SDS-PAGE loading buffer for 10 min. The eluted proteins were analyzed by Western blotting.

### 2.9. Cleavage Under Targets and Release Using Nuclease (Cut&Run)

Intact cells were bound to Concanavalin A-coated magnetic beads and permeabilized gently. The beads-bound cells were incubated with a primary antibody against the target protein (or IgG as negative control) at 4 °C overnight with gentle rotation. After washing to remove the unbound antibody, MNase (Micrococcal Nuclease) conjugated to the secondary antibody was added and incubated at 4 °C for 1 h. Nuclease digestion was initiated by shifting to 37 °C for 30 min, and the reaction was terminated with EDTA-containing buffer. Released DNA fragments were purified and worked as templated for qPCR. The Hyperactive pG-MNase CUT&RUN Assay Kit for PCR/qPCR (Vazyme Biotech, Nanjing, China) was used.

### 2.10. Cell Viability Detection

Cell Counting Kit-8 (CCK-8) kit was purchased from Beyotime Biotechnology (Shanghai, China), and the assay was performed following the kit instructions.

### 2.11. Subcutaneous Tumorigenesis Assay in Mice

The study was approved by the Ethics Committee of the School of Basic Medical Sciences, Shandong University with an ethics code ECSBMSSDU2024-2-215, approved on 5 June 2024. Six-week-old BALB/c nude mice were divided into three groups for subcutaneous tumorigenesis. The mice were injected with 1 × 107 control or ASF1B over-expressed PLC/PRF/5 cells subcutaneously. From the fourth day, a group of mice with ASF1B over-expressed cells were intraperitoneally injected with C646 at 15 mg/kg, while the control group and another ASF1B over-expression group were intraperitoneally injected with DMSO. The tumors were collected at Day 15 for HE staining, IHC (immunohistochemistry) staining and qPCR.

### 2.12. Statistical Analysis

The datasets for HCC cases within the TCGA database were analyzed by UALCAN [21]. The data were collected and shown as mean ± standard deviation (SD). Statistical analysis was performed by using the Mann–Whitney and ANOVA tests. *p* < 0.05 was considered to be statistically different.

## 3. Results

### 3.1. Profiles of Differentially Expressed lncRNAs, miRNAs, circRNAs, and mRNAs in HCC

**Figure 1:**
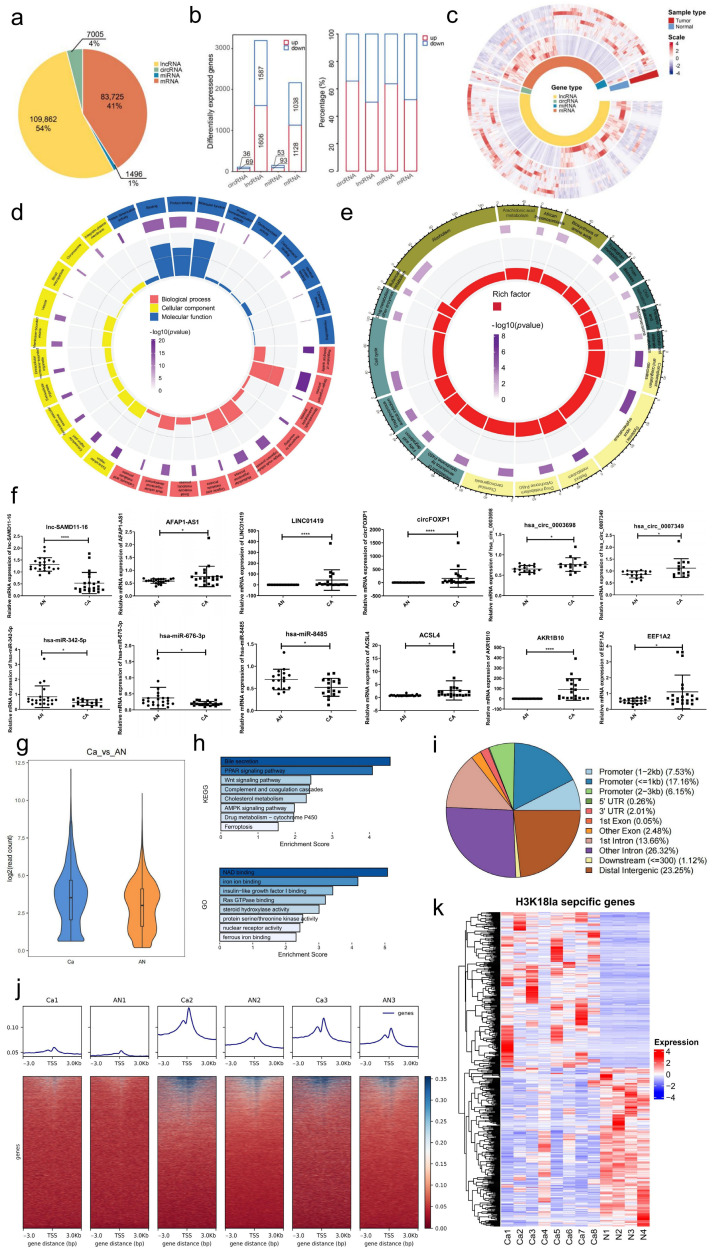
Profiles of differentially expressed RNAs and genes with differentially enriched H3K18la in HCC. (**a**) The number and percentage of all detected RNAs and the histogram of differentially expressed (DE) lncRNAs, circRNAs, miRNAs, and mRNAs; (**b**) The percentage of either up- or downregulated RNAs; (**c**) The heatmap showing the hierarchical clustering of altered lncRNAs, circRNAs, miRNAs, and mRNAs between the HCC and AN groups. Red represents upregulation, and blue represents downregulation; (**d**) GO enrichment analysis demonstrating MF, BP, and CC terms of DE mRNAs; (**e**) KEGG enrichment analysis of DE mRNAs; (**f**) The expression of selected DE lncRNA, circRNA, miRNA and mRNA in RNA-Seq were detected in the Validation cohort of HCC cancer (CA) tissue and adjacent normal (AN) tissue; (**g**) The violin plot of the average number of reads in ChIP-Seq for HCC and AN; (**h**) KEGG and GO enrichment analysis of H3K18la differentially enriched genes; (**i**) The distribution of differential peaks in the genome; (**j**) Heatmaps for H3K18la binding peaks in HCC and AN samples; (**k**) Heat maps showing expression kinetics of H3K18la-specific genes. *p* < 0.05 was considered to be statistically different. * *p* < 0.05, **** *p* < 0.0001.

In the present study, the sequencing cohort samples, including eight HCC tumor specimens and four AN tissues, were collected for high-throughput next-generation sequencing to explore the expression landscape of the whole genome. After data analysis, 109,862 (54%) lncRNAs, 7005 (4%) circRNAs, 1496 (1%) miRNAs, and 83,725 (41%) mRNAs were detected in all samples (Figure 1a). Compared to the AN group, the expression profiles showed that 1606 lncRNAs, 69 circRNAs, 93 miRNAs, and 1128 mRNAs were significantly upregulated, while 1587 lncRNAs, 36 circRNAs, 53 miRNAs, and 1038 mRNAs were significantly downregulated (*p* < 0.05) in the HCC group (Figure 1b). The percentage of up- and downregulated ncRNAs varied slightly among the groups. An overview of the dysregulation levels for all detected DE ncRNAs in HCC is shown in a heatmap, which demonstrates remarkable interpatient variation (Figure 1c). Hence, the expression landscape changes in ncRNA regulatory networks highly contributed to HCC development.

GO and KEGG enrichment analyses of DE mRNAs were performed to investigate gene functions. The GO terms covered the following three domains in GO enrichment analysis: biological processes (BP), cellular components (CC), and molecular functions (MF). Figure 1d presents the top 10 enrichments of each category. For DE mRNAs, the most enriched GO terms were single-organism process (GO ID: GO:0044700; type: BP; *p* < 0.01), multicellular organismal process (GO ID: GO:0032501; type: BP; *p* < 0.001), vesicle (GO ID: GO:0031982; type: CC; *p* < 0.001), extracellular region (GO ID: GO:0005576; type: CC; *p* < 0.001), molecular function (GO ID: GO:0003674; type: MF; *p* < 0.001), and binding (GO ID: GO:0005488; type: MF; *p* < 0.001).

The top 50 pathways from KEGG analysis were identified, which suggested the main pathways of DE mRNAs (Figure 1e). The liver is the major organ that metabolizes different chemicals, including drugs and alcohol. HCC causes liver cells to lose their function; therefore, it can influence multiple metabolic processes, such as drug metabolism, fatty acid degradation, alcoholism, and chemical carcinogenesis. Furthermore, the glycolysis/gluconeogenesis pathway, which regulates the accumulation and consumption of lactate, was also one of the most enriched. In most cases, lactate levels in HCC are upregulated, which is consistent with the KEGG analysis.

qPCR was performed to determine the expression of selected lncRNAs, circRNAs, miRNAs, and mRNAs using validation cohort samples, including 22 pairs of HCC tumors and AN tissues, to validate the high-throughput sequencing results (Figure 1f). ENSG00000235146 (lnc-SAMD11) was downregulated, and ENSG00000253898 (LINC01419) and ENSG00000272620 (AFAP1-AS1) were upregulated in HCC. Regarding circRNAs, circFOXP1, hsa_circ_0007349, and hsa_circ_0003698 were upregulated in HCC. In terms of miRNAs, hsa-miR-676-3p, hsa-miR-342-5p, and hsa-miR-8485 were downregulated in HCC. As for mRNAs, aldo-keto reductase family 1 member B10 (AKR1B10), acyl-CoA synthetase long-chain family member 4 (ACSL4), and eukaryotic elongation factor 1A2 (EEF1A2) were upregulated in HCC. These results were consistent with our sequencing data.

### 3.2. Profiles of Genes with Differentially Enriched H3K18la in HCC

The accumulation of lactate in HCC can increase protein lactylation in tumor cells and accelerate malignancy. Particularly, histone lactylation has a widespread influence on different processes, such as metabolism and cell fate. However, the research on the landscape of histone lactylation-regulated ncRNAs is lacking. The ChIP-Seq was performed in three pairs of HCC and AN samples to explore how H3K18la influences the ncRNA regulatory network in HCC. Thus, 10,582 different peaks between HCC and AN were detected, as well as 2831 down-peak-related genes and 3428 up-peak-related genes in HCC (*p* < 0.05). The reads count showed a higher level of H3K18la in HCC compared to AN (Figure 1g). The KEGG analysis of genes with different H3K18la enrichment identified that PPAR and the Wnt signaling pathway were the main pathways, while GO analysis suggested that NAD binding and iron ion binding were most enriched (Figure 1h). Furthermore, 30.84% of peaks were located in the promoter region, accounting for the highest proportion (Figure 1i). The data showed that H3K18la was more enriched in promoter regions and upstream regions of genes in HCC compared to AN (Figure 1j). An overview of the dysregulation levels for H3K18la-specific mRNAs and lncRNAs in HCC is shown in a heatmap, which demonstrates remarkable interpatient variation (Figure 1k).

### 3.3. The lncRNA-miRNA-mRNA Regulatory Network Is Influenced by Differentially Enriched H3K18la

**Figure 2:**
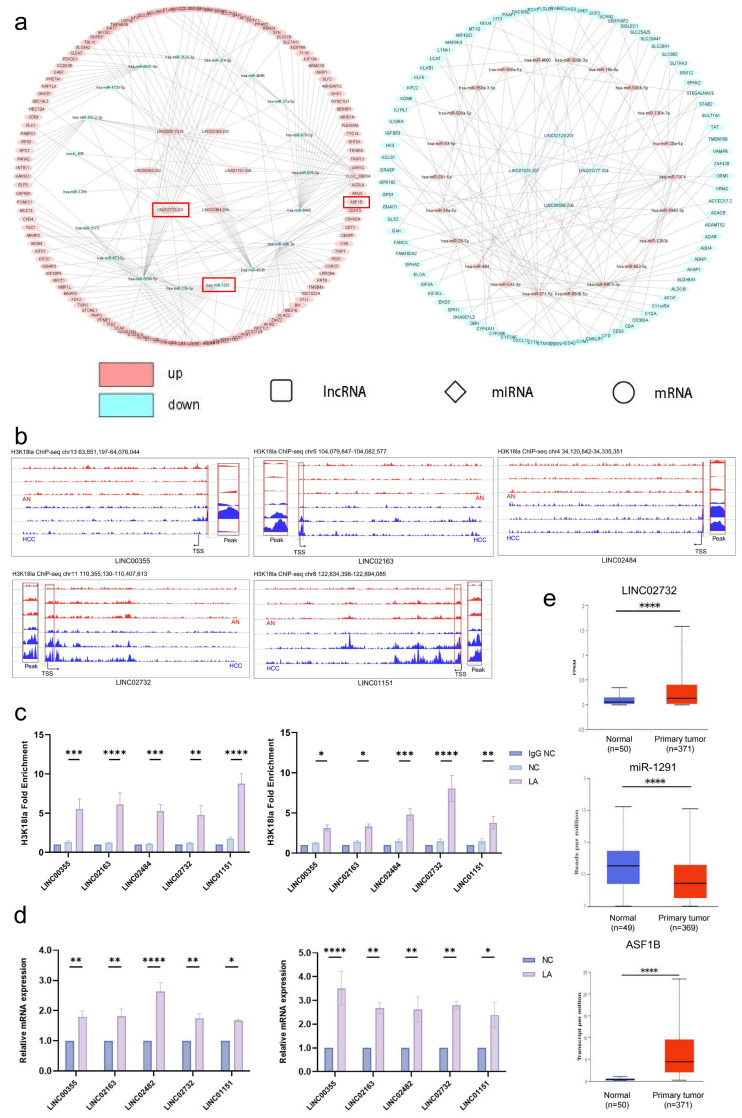
The lncRNA-miRNA-mRNA regulatory network influenced by differentially enriched H3K18la in HCC. (**a**) The network of upregulated lncRNA-downregulated miRNA-upregulated mRNA and downregulated lncRNA-upregulated miRNA-downregulated mRNA is influenced by H3K18la on the lncRNA promoter; (**b**) The H3K18la enrichment peaks on the LINC00355, LINC02163, LINC02484, LINC02732, and LINC01151 promoter region in AN and HCC; The H3K18la enrichment on the promoter (**c**) and mRNA expression (**d**) of LINC00355, LINC02163, LINC02484, LINC02732, and LINC01151 after lactate (LA) treatment in HCC cell lines Huh-7 (**left**) and PLC/PRF/5 (**right**); (**e**) The RNA expression of LINC02732, miR1291, and ASF1B in HCC and AN samples from TCGA. *p* < 0.05 indicated statistical significance. * *p* < 0.05, ** *p* < 0.01, *** *p* < 0.001, **** *p* < 0.0001.

The lncRNA-miRNA-mRNA network based on whole transcriptome sequencing was constructed first to further explore the regulatory function of lncRNAs and the influence of H3K18la on it in HCC. DE lncRNA-targeted DE miRNAs, DE miRNA-targeted DE mRNAs, and DE lncRNA-targeted DE mRNAs were analyzed to construct the changed lncRNA-miRNA-mRNA network in HCC. The H3K18la ChIP-Seq identified 6791 sites that were more enriched in HCC compared to AN and 129 sites that were located on the promoter region of different lncRNA-coding genes, which might upregulate gene expression. Combining with the ChIP-Seq results, several axes that are regulated by H3K18la on lncRNA promoter were demonstrated as ‘up-down-up’ and ‘down-up-down’ regulation networks (Figure 2a). According to the ChIP-Seq analysis, the H3K18la enrichment peaks of five lncRNAs in the ‘up-down-up’ regulation network were presented, which were all located near the transcription start site (TSS) (Figure 2b). Expression and the H3K18la site of these lncRNAs were validated in cell lines, showing that both the promoter H3K18la enrichment and expression of LINC00355, LINC02163, LINC02484, LINC02732, and LINC01151 were upregulated after 48 h with 20mM lactate (LA) treatment (Figure 2c,d). The LINC02732-miR-1291-ASF1B axis was selected for further research (highlighted with red box in Figure 2a). The ‘up-down-up’ expression of the LINC02732-miR1291-ASF1B axis in HCC was validated in TCGA data by a UALCAN platform. Compared to AN, LINC02732 and ASF1B were upregulated in HCC, while miR-1291 was downregulated in HCC (Figure 2e). As for H3K18la, ChIP-Seq showed three significant upregulated peaks on the LINC02732 promoter in HCC, which were located at +1963, 0, and −2565 to the TSS, respectively. However, no significant enriched peaks were detected on the ASF1B promoter in HCC, suggesting that H3K18la indirectly regulated ASF1B transcription by LINC02732-miR-1291.

### 3.4. H3K18la Influences LINC02732-miR-1291-ASF1B in HCC by Upregulating LINC02732 Expression

**Figure 3:**
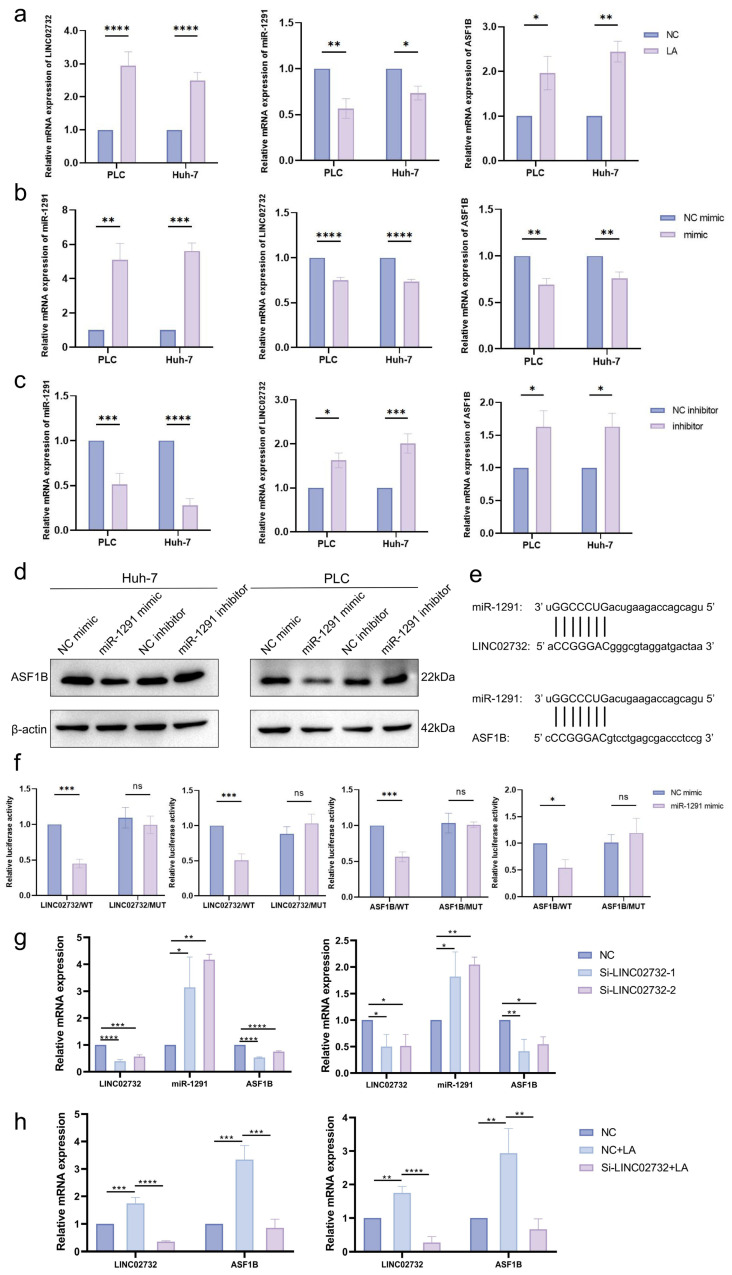
H3K18la influences LINC02732-miR1291-ASF1B in HCC by upregulating LINC02732 expression. (**a**) The RNA expression of LINC02732, miR-1291, and ASF1B in HCC cell lines after lactate (LA) treatment; The RNA expression of LINC02732, miR-1291, and ASF1B in HCC cell lines with miR-1291 mimic transfection (**b**) and miR-1291 inhibitor transfection (**c**); (**d**) The protein level of ASF1B with miR-1291 mimic/inhibitor transfection; (**e**) The complementary sequence between miR-1291 and LINC02732/ASF1B; (**f**) The relative luciferase activity of the reporter containing wild-type or mutant LINC02732/ASF1B complementary sequence with miR-1291 mimic co-transfection in Huh-7 (**left**) and PLC/PRF/5 (**right**); (**g**) The RNA expression of LINC02732, miR-1291, and ASF1B with LINC02732 knockdown in Huh-7 (**left**) and PLC/PRF/5 (**right**); (**h**) The RNA expression of LINC02732 and ASF1B with LA or LA+ Si-LINC02732-1 in Huh-7 (**left**) and PLC/PRF/5 (**right**). *p* < 0.05 indicated statistical significance. * *p* < 0.05, ** *p* < 0.01, *** *p* < 0.001, **** *p* < 0.0001. ns means not significant.

A further study was performed to validate the H3K18la-regulated LINC02732-miR1291-ASF1B axis in HCC. In HCC cell lines Huh-7 and PLC/PRF/5, lactate significantly increased the H3K18la level on the LINC02732 promoter and LINC02732 RNA expression compared to the negative control (NC) (Figure 2c,d). Moreover, the mRNA level of ASF1B was upregulated, while miR-1291 expression was downregulated (Figure 3a). Upregulating miR-1291 by miR-1291 mimic significantly decreased the RNA expression of both LINC02732 and ASF1B, while miR-1291 inhibitor increased LINC02732 and ASF1B expression, which demonstrated the molecular sponge role of miR-1291 on LINC02732 and ASF1B at the expression level (Figure 3b,c). The protein level of ASF1B was also detected in cells treated with miR-1291 mimic or inhibitor, with the trend of change being consistent with the mRNA level (Figure 3d).

A dual luciferase reporter plasmid containing wild-type miR-1291-ASF1B/LINC02732 pairing sequence was constructed, as well as the plasmid containing the mutant pairing sequence (Figure 3e). Following co-transfection, the miR-1291 mimic significantly decreased the luciferase activity of wild-type LINC02732 or ASF1B pairing sequence but had no effect on the reporter containing mutant sequence, which suggested that miR-1291 downregulated the RNA level of LINC02732 and ASF1B by binding to the target sequence (Figure 3f).

LINC02732 knockdown significantly upregulated miR-1291 and downregulated ASF1B expression (Figure 3g). Moreover, LINC02732 knockdown reversed the upregulation of ASF1B induced by LA treatment (Figure 3h).

### 3.5. ASF1B Recruits p300 to Promote H3K18la Modification and Establish Positive Feedback on LINC02732

**Figure 4:**
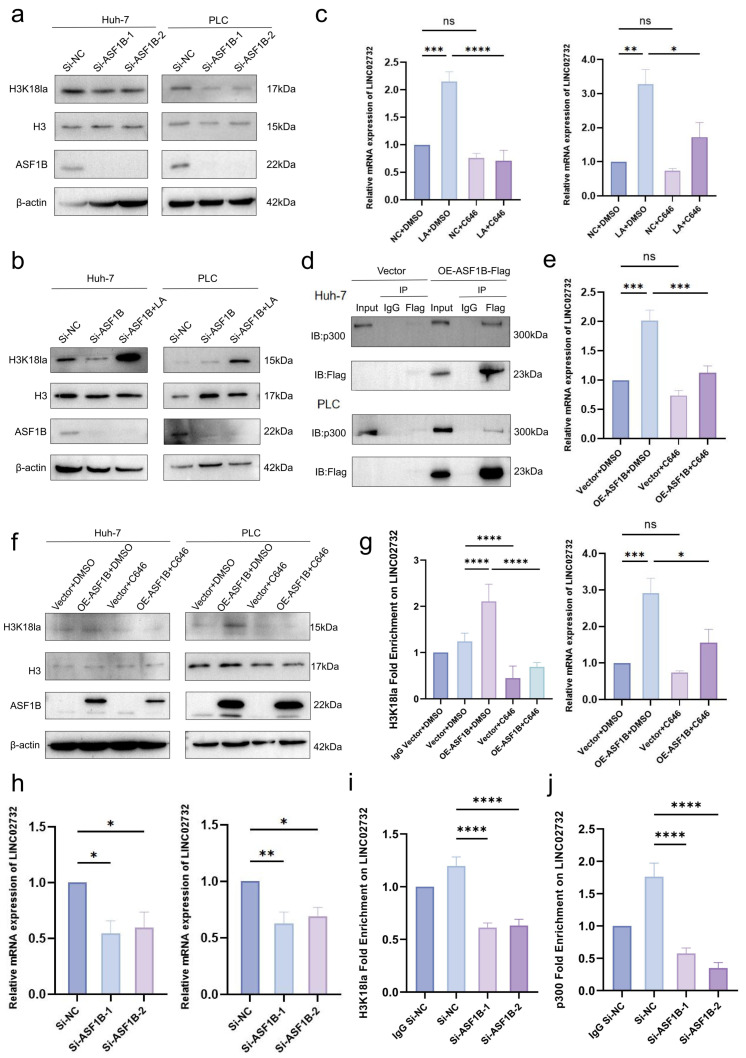
ASF1B recruits p300 to promote H3K18la modification and establish positive feedback on LINC02732. (**a**) H3K18la level after ASF1B knockdown; (**b**) Lactate (LA) treatment rescued the downregulated H3K18la level induced by ASF1B knockdown with Si-ASF1B-1; (**c**) The RNA expression of LINC02732 with LA or/and C646 in Huh-7 (**left**) and PLC/PRF/5 (**right**); (**d**) p300 in Co-IP with Flag-ASF1B pulling-down; (**e**) The RNA expression of LINC02732 with ASF1B overexpression (OE-ASF1B) and/or C646 in Huh-7 (**up**) and PLC/PRF/5 (**down**); (**f**) H3K18la level with ASF1B overexpression and/or C646; (**g**) The H3K18la enrichment on the LINC02732 promoter with ASF1B overexpression and/or C646 in Huh-7; (**h**) The RNA expression of LINC02732 with ASF1B knockdown in Huh-7 (**left**) and PLC/PRF/5 (**right**); (**i**) The H3K18la enrichment on the LINC02732 promoter with ASF1B knockdown in PLC/PRF/5; (**j**) The p300 binding enrichment on the LINC02732 promoter with ASF1B knockdown in Huh-7. *p* < 0.05 indicated statistical significance. * *p* < 0.05, ** *p* < 0.01, *** *p* < 0.001, **** *p* < 0.0001. ns means not significant.

ASF1B, functioning as a chaperone for H3-H4 histones, could facilitate multiple histone modifications, involving acetylation. However, whether ASF1B also promotes histone lactylation is unclear. The current study showed that H3K18la level was significantly downregulated after ASF1B knockdown; therefore, ASF1B also played an important role in histone lactylation (Figure 4a). Moreover, lactate restored the decrease in H3K18la caused by ASF1B knockdown (Figure 4b).

The p300 protein plays a catalytic role in histone lactylation. Treating cells with p300 inhibitor C646 significantly reversed the upregulation of LINC02732 by lactate (Figure 4c). Co-IP was performed in HCC cells that stably overexpressed ASF1B to explore whether ASF1B promoted H3K18la by recruiting p300, confirming the binding between p300 and ASF1B (Figure 4d). ASF1B overexpression significantly upregulated H3K18la, while C646 reversed the effect of ASF1B (Figure 4f).

The promoting role of ASF1B in H3K18la could form positive feedback to LINC02732. ASF1B overexpression significantly enhanced LINC02732 expression, and C646 reduced this effect (Figure 4e). The H3K18la enrichment was increased in cells overexpressing ASF1B, and the trend was reversed with C646 treatment (Figure 4g). ASF1B knockdown impaired LINC02732 expression and the H3K18la level on the LINC02732 promoter (Figure 4h, i). Furthermore, ASF1B knockdown decreased p300 enrichment on the LINC02732 promoter, suggesting that ASF1B promoted LINC02732 transcription by recruiting p300 to elevate H3K18la level (Figure 4j).

### 3.6. The ASF1B-H3K18la Positive Feedback Loop Promotes CD44 Expression and HCC Cell Stemness

**Figure 5:**
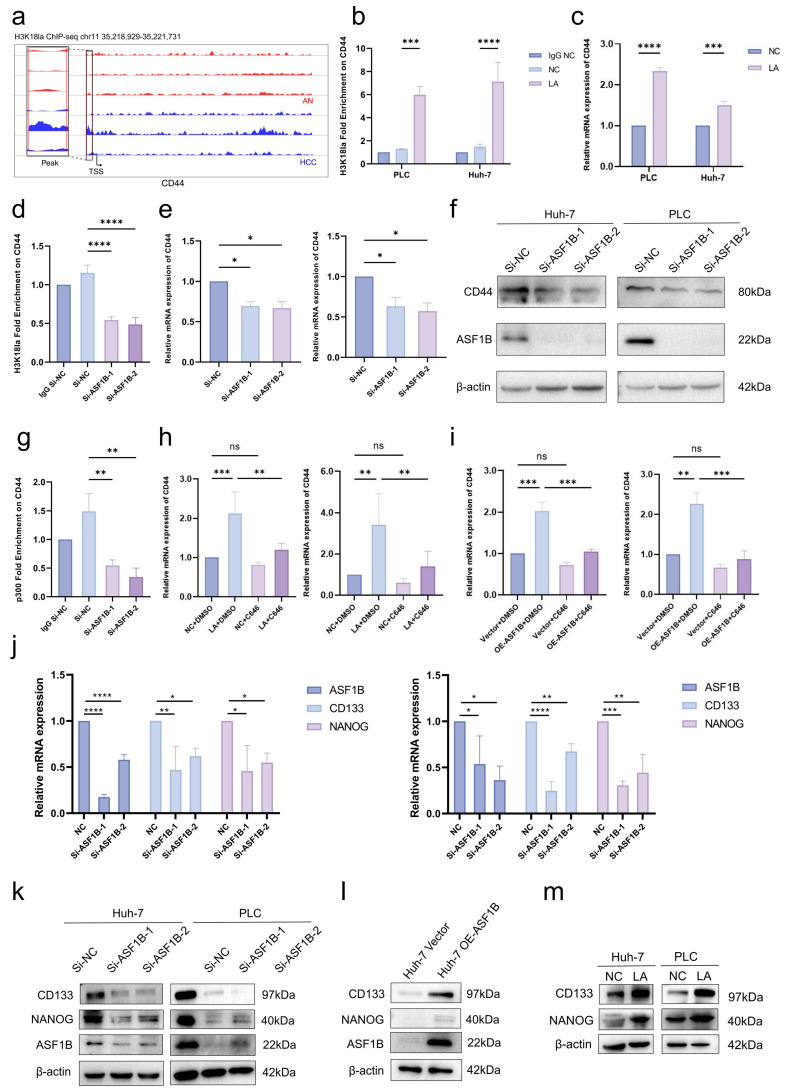
The ASF1B-H3K18la axis enhances HCC cell stemness by promoting CD44 expression. (**a**) The H3K18la enrichment peaks on the CD44 promoter region in AN and HCC; (**b**) The H3K18la enrichment on the CD44 promoter with lactate (LA) treatment; (**c**) The mRNA expression of CD44 after LA treatment; (**d**) The H3K18la enrichment on the CD44 promoter with ASF1B knockdown in PLC/PRF/5; The mRNA (**e**) and protein (**f**) expression of CD44 after ASF1B knockdown in Huh-7 (**left**) and PLC/PRF/5 (**right**); (**g**) The p300 binding enrichment on the CD44 promoter with ASF1B knockdown in Huh-7; (**h**) The mRNA expression of CD44 with LA and/or C646 in Huh-7 (**left**) and PLC/PRF/5 (**right**); (**i**) The mRNA expression of CD44 with ASF1B overexpression and/or C646 in Huh-7 (**left**) and PLC/PRF/5 (**right**); (**j**) The mRNA expression of cancer stem cell markers CD133 and NANOG after ASF1B knockdown in Huh-7 (**left**) and PLC/PRF/5 (**right**); (**k**) The protein level of ASF1B, CD133 and NANOG after ASF1B knockdown; (**l**) The protein level of ASF1B, CD133 and NANOG with ASF1B overexpression; (**m**) The protein level of CD133 and NANOG with LA treatment. *p* < 0.05 indicated statistical significance. * *p* < 0.05, ** *p* < 0.01, *** *p* < 0.001, **** *p* < 0.0001. ns means not significant.

The comprehensive analysis of mRNA sequencing and ChIP-Seq indicated a significant increase in both mRNA level and promoter H3K18la enrichment of CD44 in HCC (−503 to TSS; Figure 5a). CD44, a recognized stem cell marker, plays an important role in cell proliferation. Further research validated that lactate elevated H3K18la level on its promoter region and enhanced CD44 transcription in HCC cells (Figure 5b,c). ASF1B knockdown reduced H3K18la enrichment and CD44 expression, demonstrating that ASF1B-H3K18la positive feedback promoted CD44 transcription (Figure 5d–f). ASF1B knockdown impaired the binding of p300 to the CD44 promoter (Figure 5g). C646 significantly reversed the upregulation of CD44 by lactate (Figure 5h). ASF1B overexpression increased CD44 expression, and the effect was reversed by C646 (Figure 5i).

ASF1B knockdown markedly downregulated cancer stem cell markers CD133 and NANOG at both mRNA and protein levels (Figure 5j,k). ASF1B overexpression obviously upregulated the expression of these two markers (Figure 5l). LA treatment elevated protein expression of CD133 and NANOG (Figure 5m). These findings indicated that ASF1B and H3K18la promoted HCC cell stemness via CD44.

### 3.7. The ASF1B-H3K18la Axis Promotes HCC Cell Proliferation In Vitro and In Vivo

**Figure 6:**
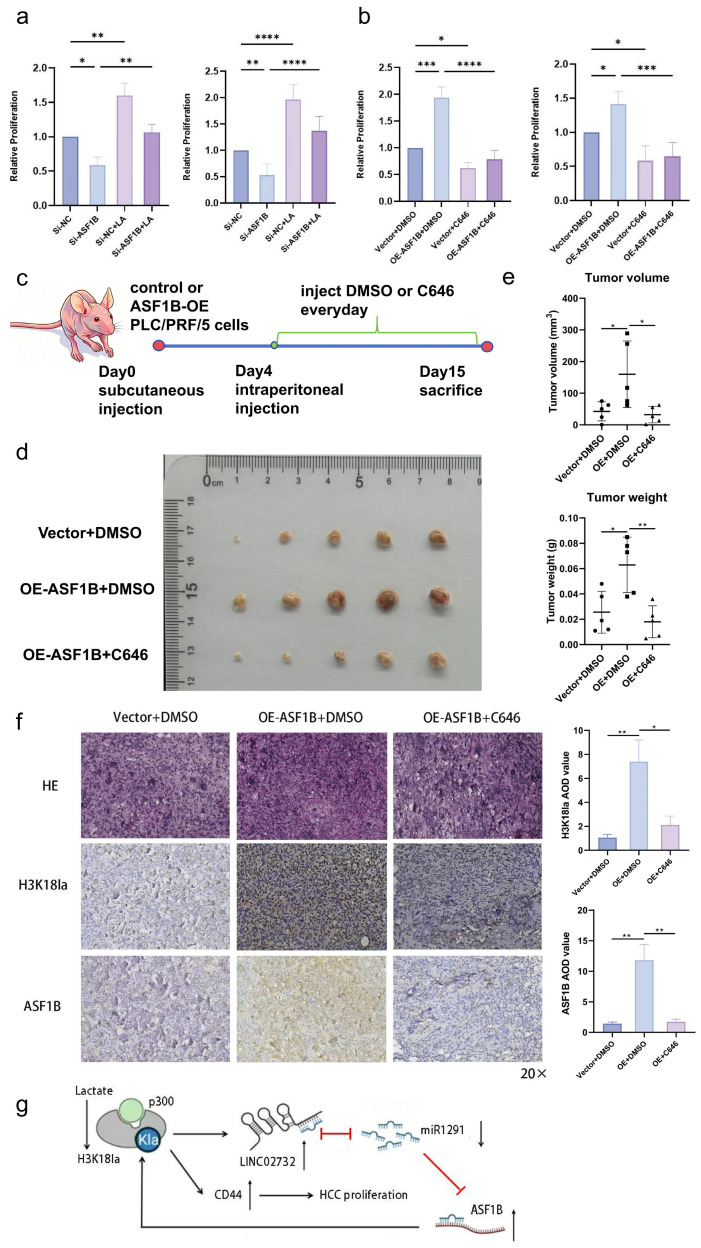
The ASF1B-H3K18la axis promotes HCC cell proliferation in vitro and in vivo. (**a**) CCK8-detecting cell viability with Si-ASF1B-1 and/or lactate (LA) in Huh-7 (**left**) and PLC/PRF/5 (**right**); (**b**) CCK8-detecting cell viability with ASF1B overexpression and/or C646 in Huh-7 (**left**) and PLC/PRF/5 (**right**); (**c**) The flow diagram of the mouse experiment; (**d**) The tumors in nude mice generated by vector-PLC/PRF/5 and lenti-ASF1B-PLC/PRF/5 cells with DMSO or C646 intraperitoneal injection; (**e**) Tumor weight and volume; (**f**) The ASF1B and H3K18la levels in tumors, 20× HE and IHC staining (**left**) and average optical density (AOD) analysis (**right**); (**g**) H3K18la-LINC02732-miR-1291-ASF1B positive feedback loop by recruiting p300 in HCC proliferation. *p* < 0.05 indicated statistical significance. * *p* < 0.05, ** *p* < 0.01, *** *p* < 0.001, **** *p* < 0.0001.

CCK8 was performed to explore the biological effect of ASF1B-H3K18la, showing that ASF1B knockdown significantly impaired the enhancement of cell proliferation by lactate (Figure 6a). ASF1B overexpression promoted cell proliferation, and C646 reversed its effect (Figure 6b).

The H3K18la-ASF1B axis was further validated in BALB/c nude mice (Figure 6c). Compared to mice receiving a control PLC/PRF/5 cell injection subcutaneously, mice injected with ASF1B-overexpressing cells developed tumors more rapidly. Furthermore, C646 significantly inhibited the proliferation-promoting effect of ASF1B (Figure 6d,e). IHC demonstrated that the tumors developed from ASF1B-overexpressing cells showed higher ASF1B expression and H3K18la level, and C646 significantly decreased the H3K18la and ASF1B level (Figure 6f).

The results suggested that H3K18la influenced the LINC02732-miR-1291-ASF1B axis, and ASF1B recruited p300 to facilitate H3K18la enrichment on LINC02732 and CD44 promoter, which formed a positive feedback loop and contributed to HCC proliferation (Figure 6g).

## 4. Discussion

The role of histone lactylation in coding-gene transcription has been extensively studied; however, research on how it influences ncRNA expression and its regulatory network is still lacking. The current study focused on the representative histone lactylation site H3K18la and performed ChIP-Seq and transcriptome sequencing in HCC and AN clinical samples. lncRNA, miRNA, and mRNA sequencing were used to jointly construct the ncRNA regulatory network that is differentially expressed in HCC, in which every lncRNA-miRNA-mRNA axis changed in either ‘up-down-up’ or ‘down-up-down’ fashion due to endogenous competitive binding between miRNA and lncRNA/mRNA. The combined analysis of H3K18la ChIP-Seq and RNA-Seq further showed that lncRNA was influenced by H3K18la. Additionally, overlapping with the network mentioned above, it finally built an H3K18la-regulating lncRNA-miRNA-mRNA network. Among the axes, the LINC02732-miR-1291-ASF1B was promising in terms of HCC development promotion, and its expression was validated using TCGA data.

In this ncRNA regulatory axis, LINC02732 is a novel ncRNA with unknown regulatory functions, while miR-1291 exerts an antitumor effect in some human cancers. miR-1291, which expression is downregulated in kidney and esophagus cancer, inhibits tumorigenesis and sensitizes pancreatic cancer cells to chemotherapy [22,23,24,25]. In colon cancer, miR-1291 serves as an antitumor agent by inhibiting doublecortin-like kinase 1 (DCLK1) expression [26]. Our transcriptome sequencing discovered that HCC tissue had a lower expression of miR-1291 compared to AN, and the trend was validated in 369 HCC and 49 AN samples (TCGA data). Hence, miR-1291 also played an antitumor role in HCC as in other cancers. ASF1B promotes the growth of various cancers. ASF1B can promote cervical cancer by CDK9 stabilization and promote gastric cancer by modulating H2AC20 [15,27]. In HCC, ASF1B was positively correlated with Treg cell infiltration and inhibitory immune checkpoints in exhausted T cells, which may serve as a potential immunotherapeutic target [28]. Our study demonstrated that ASF1B was indirectly regulated by H3K18la modulation via the ‘up-down-up’ ncRNA axis—LINC02732-miR-1291-ASF1B, in which H3K18la enrichment was found not on the ASF1B promoter but on the LINC02732 promoter.

Further study revealed a novel role of ASF1B in histone lactylation besides its canonical role as a histone chaperone. ASF1B could recruit p300, a lactyltransferase catalyzing histone lactylation, to facilitate H3K18la modification. ASF1B could bind to p300 and significantly elevate LINC02732 expression, as well as p300 and H3K18la enrichment on the LINC02732 promoter. Taken together, the ASF1B-H3K18la-LINC02732 positive feedback loop was validated. Moreover, ASF1B promoted cell proliferation by influencing the expression of other oncogenes via H3K18la. ChIP-Seq and mRNA Seq showed that CD44, a stem cell marker, was also upregulated by H3K18la in HCC. Further research in HCC cells revealed that CD44 expression was influenced by ASF1B-H3K18la. CD44 worked as the effector of the H3K18la-LINC02732-miR-1291-ASF1B axis and elevated HCC cell stemness, increasing HCC cell proliferation.

## 5. Conclusions

In conclusion, the accumulation of lactate in the HCC tumor microenvironment upregulates global H3K18la, which influences not only the coding-gene transcription but also the noncoding-gene transcription, therefore changing the ncRNA regulatory network. In the ‘up-down-up’ axis LINC02732-miR-1291-ASF1B, LINC02732 was upregulated by H3K18la on its promoter. ASF1B recruits p300 to catalyze H3K18la modification, forming a positive feedback loop. ASF1B-H3K18la also increases the transcription of CD44, eventually promoting HCC cell proliferation.

## Data Availability

The original data presented in the study are openly available in Sequence Read Archive (SRA) PRJNA808183.

## Supplementary Materials

The following supporting information can be downloaded at: https://www.mdpi.com/article/10.3390/cells15100952/s1, Table S1: Primers used in qPCR.

## Abbreviations

The following abbreviations are used in this manuscript:

AN	adjacent normal
ASF1	Anti-silencing Function 1
BP	biological processes
CC	cellular components
Co-IP	Co-Immunoprecipitation
Cut&Run	Cleavage Under Targets and Release Using Nuclease
DE	differentially expressed
HCC	hepatocellular carcinoma
IHC	immunohistochemistry
GO	Gene Ontology
KEGG	Kyoto Encyclopedia of Genes and Genomes
Kla	lysine lactylation
lncRNA	long noncoding RNA
miRNA	microRNA
MF	molecular functions
NC	negative control
ncRNA	noncoding RNA
qPCR	quantitative PCR
TSS	transcription start site

## References

[1] Vogel A., Meyer T., Sapisochin G., Salem R., Saborowski A. (2022). Hepatocellular carcinoma. Lancet.

[2] Koshy A. (2025). Evolving Global Etiology of Hepatocellular Carcinoma (HCC): Insights and Trends for 2024. J. Clin. Exp. Hepatol..

[3] Zhang D., Tang Z., Huang H., Zhou G., Cui C., Weng Y., Liu W., Kim S., Lee S., Perez-Neut M. (2019). Metabolic regulation of gene expression by histone lactylation. Nature.

[4] Yang K., Fan M., Wang X., Xu J., Wang Y., Tu F., Gill P.S., Ha T., Liu L., Williams D.L. (2022). Lactate promotes macrophage HMGB1 lactylation, acetylation, and exosomal release in polymicrobial sepsis. Cell Death Differ..

[5] Peng X., Du J. (2025). Histone and non-histone lactylation: Molecular mechanisms, biological functions, diseases, and therapeutic targets. Mol. Biomed..

[6] Chen J., Huang Z., Chen Y., Tian H., Chai P. (2025). Lactate and lactylation in cancer. Signal Transduct. Target. Ther..

[7] Chu X., Di C., Chang P., Li L., Feng Z., Xiao S., Yan X., Xu X., Li H., Qi R. (2021). Lactylated Histone H3K18 as a Potential Biomarker for the Diagnosis and Predicting the Severity of Septic Shock. Front. Immunol..

[8] Li W., Zhou C., Yu L., Hou Z., Liu H., Kong L., Xu Y., He J., Lan J., Ou Q. (2024). Tumor-derived lactate promotes resistance to bevacizumab treatment by facilitating autophagy enhancer protein RUBCNL expression through histone H3 lysine 18 lactylation (H3K18la) in colorectal cancer. Autophagy.

[9] Ji Y., Xu Z., Tang L., Huang T., Mu X., Ni C., Tang B., Lu H., Zhang C., Yang S. (2025). O-GlcNAcylation of YBX1 drives a glycolysis-histone lactylation feedback loop in hepatocellular carcinoma. Cancer Lett..

[10] Yu Y., Li Y., Zhou L., Cheng X., Gong Z. (2024). Hepatic stellate cells promote hepatocellular carcinoma development by regulating histone lactylation: Novel insights from single-cell RNA sequencing and spatial transcriptomics analyses. Cancer Lett..

[11] Cai J., Song L. (2024). Targeting SRSF10 might inhibit M2 macrophage polarization and potentiate anti-PD-1 therapy in hepatocellular carcinoma. Cancer Commun..

[12] Mousson F., Ochsenbein F., Mann C. (2007). The histone chaperone Asf1 at the crossroads of chromatin and DNA checkpoint pathways. Chromosoma.

[13] Cote J.M., Kuo Y.M., Henry R.A., Scherman H., Krzizike D.D., Andrews A.J. (2019). Two factor authentication: Asf1 mediates crosstalk between H3 K14 and K56 acetylation. Nucleic Acids Res..

[14] Lee K.Y., Im J.S., Shibata E., Dutta A. (2017). ASF1a Promotes Non-homologous End Joining Repair by Facilitating Phosphorylation of MDC1 by ATM at Double-Strand Breaks. Mol. Cell.

[15] Liu X., Song J., Zhang Y., Wang H., Sun H., Feng X., Hou M., Chen G., Tang Q., Ji M. (2020). ASF1B promotes cervical cancer progression through stabilization of CDK9. Cell Death Dis..

[16] Kim J.H., Youn Y., Lee J.C., Kim J., Ryu J.K., Hwang J.H. (2022). Downregulation of ASF1B inhibits tumor progression and enhances efficacy of cisplatin in pancreatic cancer. Cancer Biomark. Sect. A Dis. Markers.

[17] Chen Y., Zhou W., Gong Y., Ou X. (2023). Identification of ASF1B as a prognostic marker for liver cancer by meta-analysis and its immune value revealed by a comprehensive pan-cancer analysis of 33 human cancers. Prz. Gastroenterol..

[18] Dong M., Zhang Y., Chen M., Tan Y., Min J., He X., Liu F., Gu J., Jiang H., Zheng L. (2024). ASF1A-dependent P300-mediated histone H3 lysine 18 lactylation promotes atherosclerosis by regulating EndMT. Acta Pharm. Sin. B.

[19] Anastasiadou E., Jacob L.S., Slack F.J. (2018). Non-coding RNA networks in cancer. Nat. Rev. Cancer.

[20] Peng W.X., Koirala P., Mo Y.Y. (2017). LncRNA-mediated regulation of cell signaling in cancer. Oncogene.

[21] Chandrashekar D.S., Karthikeyan S.K., Korla P.K., Patel H., Shovon A.R., Athar M., Netto G.J., Qin Z.S., Kumar S., Manne U. (2022). UALCAN: An update to the integrated cancer data analysis platform. Neoplasia.

[22] Yamasaki T., Seki N., Yoshino H., Itesako T., Yamada Y., Tatarano S., Hidaka H., Yonezawa T., Nakagawa M., Enokida H. (2013). Tumor-suppressive microRNA-1291 directly regulates glucose transporter 1 in renal cell carcinoma. Cancer Sci..

[23] Luo H., Guo W., Wang F., You Y., Wang J., Chen X., Wang J., Wang Y., Du Y., Chen X. (2015). miR-1291 targets mucin 1 inhibiting cell proliferation and invasion to promote cell apoptosis in esophageal squamous cell carcinoma. Oncol. Rep..

[24] Tu M.J., Pan Y.Z., Qiu J.X., Kim E.J., Yu A.M. (2016). MicroRNA-1291 targets the FOXA2-AGR2 pathway to suppress pancreatic cancer cell proliferation and tumorigenesis. Oncotarget.

[25] Tu M.J., Duan Z., Liu Z., Zhang C., Bold R.J., Gonzalez F.J., Kim E.J., Yu A.M. (2020). MicroRNA-1291-5p Sensitizes Pancreatic Carcinoma Cells to Arginine Deprivation and Chemotherapy through the Regulation of Arginolysis and Glycolysis. Mol. Pharmacol..

[26] Wang J., Yokoyama Y., Hirose H., Shimomura Y., Bonkobara S., Itakura H., Kouda S., Morimoto Y., Minami K., Takahashi H. (2022). Functional assessment of miR-1291 in colon cancer cells. Int. J. Oncol..

[27] Zhao M., Zhang J., He Y., You C. (2025). ASF1B promotes gastric cancer progression by modulating H2AC20 and activating PI3K/AKT and ERK1/2 pathways. Front. Pharmacol..

[28] Zhang S., Xu L., Feng J., Tan D., Zhu Y., Hou J., Li W., Lv K., Wang W., Jiang L. (2022). ASF1B is a Promising Prognostic Biomarker and Correlates With Immunotherapy Efficacy in Hepatocellular Carcinoma. Front. Genet..

