# Supplementary material for: A Feedback Loop Driven by H3K18la and ASF1B via the LINC02732-miR-1291 Axis Promotes Hepatocellular Carcinoma Proliferation
Source: Cells. 2026 May 21;15(10):952. doi: 10.3390/cells15100952 (PMC13204551; doi:10.3390/cells15100952)
Supplement: Supplementary file 1 [file cells-15-00952-s001.zip › cells-4253387-supplementary.pdf]

Table S1. Primers used in qPCR

Primer name	Sequence (5'to3')
circ_0003698 F	GAGTGCTCAGCTGTTCTCTG
circ_0003698 R	CATCGATGTACAGGGCCTCT
circ_0007349 F	ATTTCAAAGAACTGCCCGCC
circ_0007349 R	TCTGAGGGCATAATACAGTCACA
circFOXP1 F	ACAGCTCTCAGTCCACACTC
circFOXP1 R	TGAAGCTGCAACTGTTCCTG
U6	CTCGCTTCGGCAGCACA
miR-342-5p	AGGGGTGCTATCTGTGATTGA
miR-676-3p	CTGTCCTAAGGTTGTTGAGTT
miR-8485	CACACACACACACACACGTAT
miR-1291	CCTGACTGAAGACCAGCAGT
ACSL4 F	CATCCCTGGAGCAGATACTCT
ACSL4 R	TCACTTAGGATTTCCCTGGTCC
AKR1B10 F	TCAGAATGAACATGAAGTGGGG
AKR1B10 R	TGGGCCACAACTTGCTGAC
EEF1A2 F	GAAGACCCACATCAACATCGT
EEF1A2 R	CTCCGCATTTGTAGATGAGGTG
lnc-SAMD11-16 F	TGAACTTCCGTACACAGCCC
lnc-SAMD11-16 R	GAGAACAACGCAGCAAAGCA
LINC01419 F	GACACGCCACCTTAAGAGCT
LINC01419 R	CGGTTCTCCTGCTGGTTGAT
AFAP1-AS1 F	TGCAACTGCGTGTTTACTGC
AFAP1-AS1 R	GCTGAGACCGCTGAGAACTT
GAPDH F	ACAACTTTGGTATCGTGGAAGG
GAPDH R	GCCATCACGCCACAGTTTC
LINC02732 F	ATTCCTTTTCCTCAGCCGGG
LINC02732 R	CTCAAATCAACAGGCCGCAC
LINC02163 F	GCAAAACCACGACACACTGG
LINC02163 R	AGGCTGAAGGAGAAGAAATGCA
LINC00355 F	GCTGTTCACTCTGGGTCTCC
LINC00355 R	CAGACAAATGGAGGGCGAGT
LINC01151 F	TGAGACAGCAAGACCAACCC
LINC01151 R	TGCAATCCTCCCCTCTGGTA
LINC02484 F	TCTAAGGGCTGCATGACTCC
LINC02484 R	TGTGAGGATTGGGAGCGAAA
LINC00355chip F	CACACCCACCCGGAACTC
LINC00355chip R	GAGGAGGTGTGGAGGGAGA
LINC02163chip F	GATGAACCCGGTACCTCAGA
LINC02163chip R	CTTCAGGAGGAGCCAAGATG
LINC01151chip F	CAGGCAGATGTACCCAGGAT
LINC01151chip R	CAGCTTGCAAAAGCAATGAA

LINC02732chip F	CCGTGATTACACCACTGCAC
LINC02732chip R	AGTTGGGTTTGGACCATGAA
LINC02484chip F	GTGGGAGACTCAGGCATAGC
LINC02484chip R	GGCAATGAGGGACTTAGCAC
ASF1B F	TCCGGTTCGAGATCAGCTTC
ASF1B R	GTCGGCCTGAAAGACAAACA
CD44 F	CTGCCGCTTTGCAGGTGTA
CD44 R	CATTGTGGGCAAGGTGCTATT
CD44 CHIP F	TCCCAGAGAAGTGCCATGTT
CD44 CHIP R	CCTCTTGTAGCTCCTCCCTG
